# TRACMIT: An effective pipeline for tracking and analyzing cells on micropatterns through mitosis

**DOI:** 10.1371/journal.pone.0179752

**Published:** 2017-07-26

**Authors:** Olivier Burri, Benita Wolf, Arne Seitz, Pierre Gönczy

**Affiliations:** 1 Bioimaging and Optics Platform (BIOP), School of Life Sciences, Swiss Federal Institute of Technology (EPFL), Lausanne, Switzerland; 2 Swiss Institute for Experimental Cancer Research (ISREC), School of Life Sciences, Swiss Federal Institute of Technology (EPFL), Lausanne, Switzerland; Institut de Genetique et Developpement de Rennes, FRANCE

## Abstract

The use of micropatterns has transformed investigations of dynamic biological processes by enabling the reproducible analysis of live cells using time-lapse fluorescence microscopy. With micropatterns, thousands of individual cells can be efficiently imaged in parallel, rendering the approach well suited for screening projects. Despite being powerful, such screens remain challenging in terms of data handling and analysis. Typically, only a fraction of micropatterns is occupied in a manner suitable to monitor a given phenotypic output. Moreover, the presence of dying or otherwise compromised cells complicates the analysis. Therefore, focusing strictly on relevant cells in such large time-lapse microscopy dataset poses interesting analysis challenges that are not readily met by existing software packages. This motivated us to develop an image analysis pipeline that handles all necessary image processing steps within one open-source platform to detect and analyze individual cells seeded on micropatterns through mitosis. We introduce a comprehensive image analysis pipeline running on Fiji termed TRACMIT (pipeline for TRACking and analyzing cells on micropatterns through MITosis). TRACMIT was developed to rapidly and accurately assess the orientation of the mitotic spindle during metaphase in time-lapse fluorescence microscopy of human cells expressing mCherry::histone 2B and plated on L-shaped micropatterns. This solution enables one to perform the entire analysis from the raw data, avoiding the need to save intermediate images, thereby decreasing data volume and thus reducing the data that needs to be processed. We first select micropatterns containing a single cell and then identify anaphase figures in the time-lapse recording. Next, TRACMIT tracks back in time until metaphase, when the angle of the mitotic spindle with respect to the micropattern is assessed. We designed the pipeline to allow for manual validation of selected cells with a simple user interface, and to enable analysis of cells plated on micropatterns of different shapes. For ease of use, the entire pipeline is provided as a series of Fiji/ImageJ macros, grouped into an ActionBar. In conclusion, the open source TRACMIT pipeline enables high-throughput analysis of single mitotic cells on micropatterns, thus accurately and efficiently allowing automatic determination of spindle positioning from time-lapse recordings.

## Introduction

Dynamic events that occur during mitosis, including spindle positioning, can be monitored faithfully using time-lapse microscopy. Spindle positioning is fundamental for the correct orientation of the axis of cell division during animal development and tissue homeostasis [1–3]. Forward genetic and functional genomic screens conducted in invertebrate model systems have led to the identification of components that proved to be broadly required for spindle positioning, including in human cells [1–3]. By contrast, fewer screens have been conducted directly in mammalian cells to identify genes important for this process [4], and none relied on monitoring of live cells. As a result, it is likely that the mechanisms governing spindle positioning in mammalian systems are not completely understood. To fill this gap, we designed and executed an siRNA-based screen for novel regulators of metaphase spindle positioning in human cells using time-lapse microscopy (Wolf et al., manuscript in preparation).

Although several image analysis algorithms have been developed to detect and monitor mitotic chromosomes in live cell imaging experiments [5–9], they were not well suited to analyze in an efficient manner this particular dataset, where single cells dividing on micropatterns had to be spotted and tracked. Therefore, to be able to readily analyze the live imaging data set from the siRNA-based screen, we developed a comprehensive image analysis pipeline termed TRACMIT (pipeline for TRACking and analyzing cells on micropatterns through MITosis), which is reported hereafter. The files needed for installation and use of TRACMIT have been deposited on GitHub, https://github.com/lacan/TRACMIT and a demo dataset has been made available on ZENODO with the following https://doi.org/10.5281/zenodo.232218

## Results and discussion

### Screening data set

**Fig 1:**
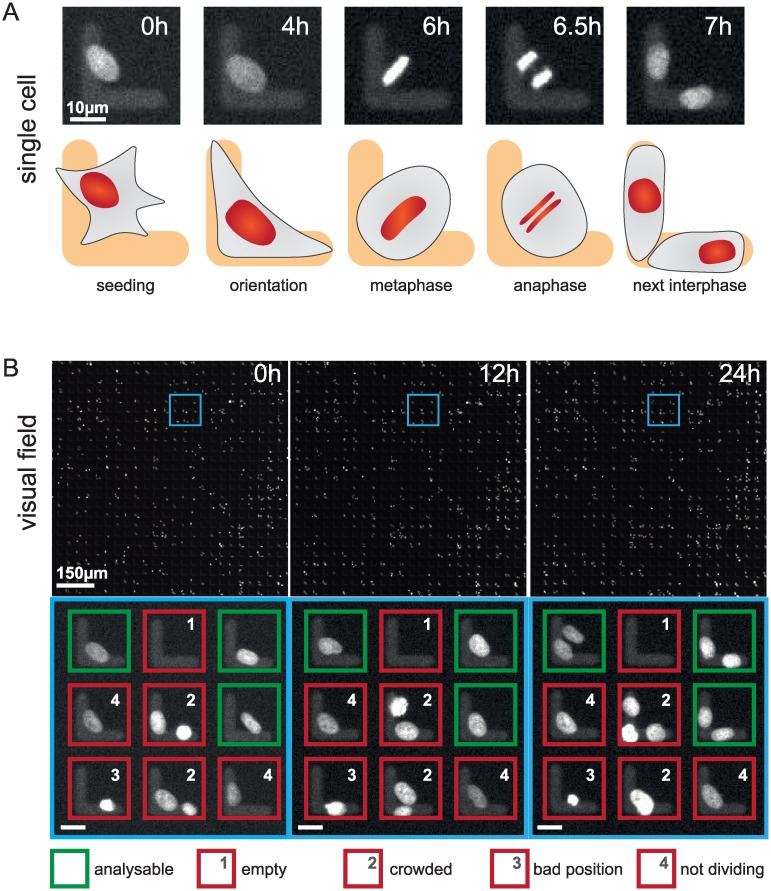
Data set for TRACMIT. **A:** Example of single HeLa cell expressing mCherry::histone 2B, grown on an L-shaped micropattern coated with fibronectin Alexa fluor 555, and monitored using time-lapse microscopy. Top: raw data from time-lapse microscopy experiment; time is indicated in hours; bottom: corresponding schematic representations. Note that the contours of the cell are shown in the schematics solely for illustration purposes and are not monitored experimentally. After seeding, the interphase cell becomes oriented on the micropattern such that the nucleus is positioned centrally between the two arms of the L. Upon cell rounding in mitosis, the metaphase plate is positioned with an angle of ~45° with respect to the arms of the L-shaped micropattern. **B:** Example of visual field of cells grown in a 96-well plate coated with L-shaped micropatterns, monitored using time-lapse microscopy; time is indicated in hours. The regions highlighted with the blue insets in the lower magnification views are enlarged below to illustrate the different classes of cells that must be discriminated by TRACMIT: green (analyzable): cell correctly positioned on the micropattern and dividing during the recording; red [1], empty: no cell on the micropattern; red [2], crowded: more than one cell on the micropattern; red [3], bad position: cell on one arm of the L; red [4], not dividing: cell not dividing during the recording. Scale bar in insets = 10μm.

We designed a screen to identify novel regulators of spindle positioning in human cells using siRNAs (Wolf et al., manuscript in preparation). Briefly, to follow division of HeLa cells on L-shaped fibronectin micropatterns by fluorescence microscopy, chromosomes are marked with mCherry::histone 2B and fibronectin with Alexa 555. In control conditions, interphase cells are typically positioned at the intersection of the two arms of the L, such that spindle positioning then occurs in a stereotyped manner during mitosis, with the metaphase plate positioned at an average angle of 45° from the two arms of the L [10] (Fig 1A). Overall, thirty-two 96-well plates containing >6000 micropatterns per well were imaged in the screen, capturing two visual fields in each well, corresponding to ~1150 micropatterns per well, every 8 minutes, and this during 24 hours (see Fig 1B, as well as S1 Movie for an example of one visual field). Both mCherry::histone 2B and Alexa 555 signals were recorded in the same channel in order to accelerate the frame rate and diminish bleaching. Overall, 3,072 wells were imaged in this manner, yielding 12.8 Tb of data.

Although we imaged a total of >3.5 million L-shaped micropatterns during the screen, only a fraction of them contained cells that could be analyzed for spindle positioning (Fig 1B, green boxes, “analyzable”). Indeed, we found that only some micropatterns were covered by exactly one cell, whereas others were either devoid of cells or had more than one (Fig 1B, “empty” and “crowded”), as had been observed in other circumstances with such micropatterns [11]. Furthermore, we found that some cells positioned themselves on only one arm of the L-shaped micropattern, instead of being at the intersection of the two arms (Fig 1B, “bad position”). In addition, some correctly positioned single cells did not divide during the span of the recording (Fig 1B, “not dividing”). Because of the above considerations, ~5% of micropatterns can effectively be analyzed for spindle positioning in these experiments (Fig 1B, green boxes).

### Pipeline development

In order to streamline the automatic analysis of spindle positioning phenotypes in such a large-scale time-lapse microscopy screening setting, we sought to develop a pipeline that could achieve the following four interconnected goals. First, recognize those micropatterns that contain one and only one cell that is correctly positioned during interphase. Second, recognize whether this cell undergoes mitosis during the recording. Third, in such a cell, determine the angle of the metaphase spindle with respect to the arms of the L. Furthermore, we aimed at developing an analysis pipeline in which all steps could be performed within the same software package in an efficient manner despite large image datasets. Finally, we wanted the solution to be widely accessible and easily adaptable, leading us to develop it as an open source platform.

**Fig 2:**
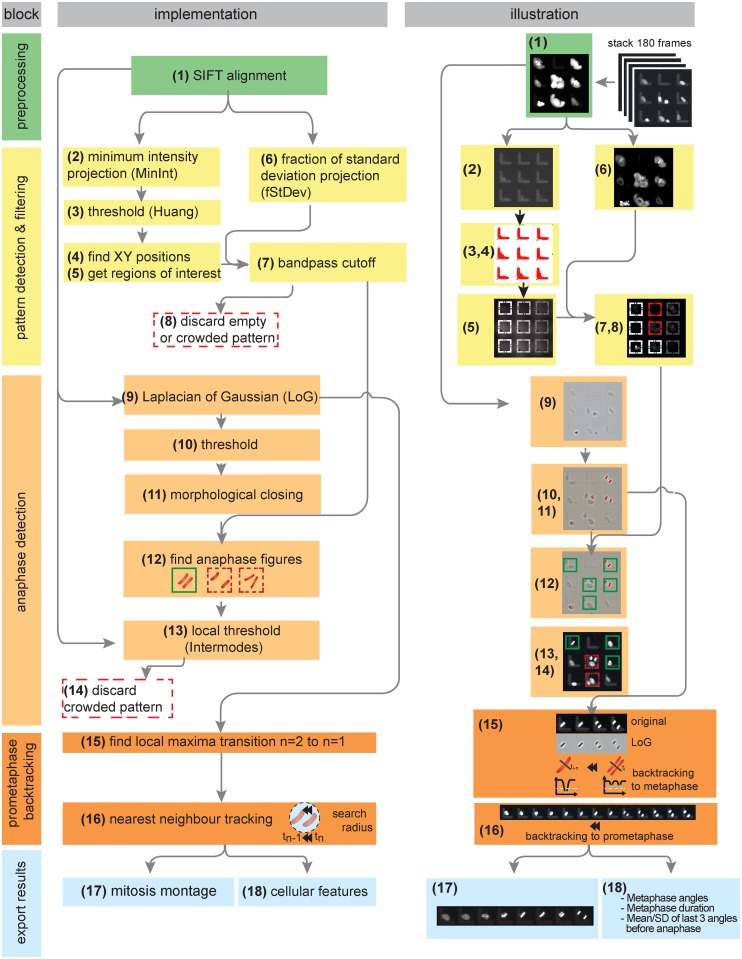
TRACMIT flowchart. Five blocks that characterize TRACMIT (preprocessing, pattern detection & filtering, anaphase detection, prometaphase backtracking, export results) are shown, together with the steps that they contain (numbered 1 through 18), as well as their computer implementation (left) and an illustration thereof (right). See text for details.

To meet these requirements, we developed a detection and analysis pipeline written as a series of ImageJ/Fiji [12, 13] macros packaged into an ActionBar [14] for ease of use. TRACMIT consists of five consecutive blocks that encompass 18 steps, which are described hereafter, illustrated graphically in Fig 2 and described also step by step in the accompanying user manual (S1 document); workflow availability and requirements of TRACMIT are summarized in S2 document. Although developed initially for analyzing cells in 96-well plates imaged with a 10x objective, TRACMIT can be adapted for single condition experiments and for imaging with other lenses by modifying parameters using the "Parameter Wizards" (S1 document).

**Fig 3:**
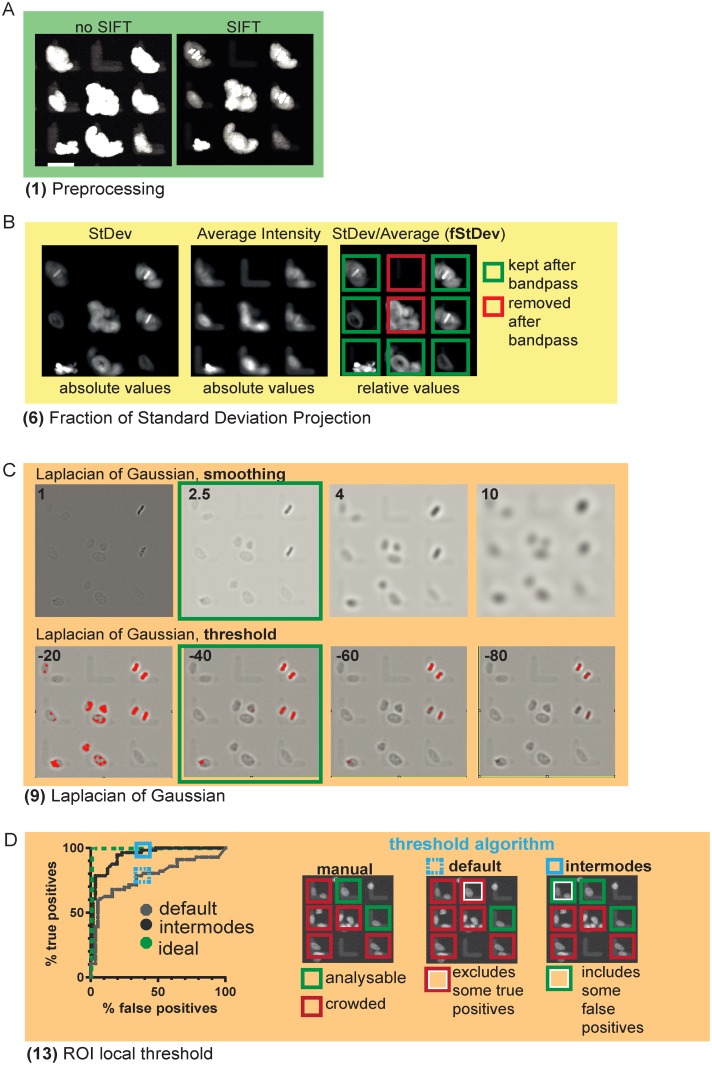
Assessment of key steps implemented in TRACMIT. **A:** Comparison of frames aligned without SIFT (left) or using SIFT (right). Note tighter area with the latter. In this panel, as well as in panels B and C, the insets correspond to those shown in Fig 2, step 1. **B:** Illustration of Fig 2, steps 6, 7 and 8, exclusion of micropatterns that are empty or contain more than one cell. Shown are z-projections of 180 frames. StDev: standard deviation of pixel intensities, used to determine cell movement, thereby reflecting the number of cells on the pattern. Average pixel intensity (middle panel): used as denominator in order to transform absolute StDev values into relative values (right panel). In this way a common bandpass threshold can be applied on ROIs with highly variable fluorescence intensities, and thus StDevs. **C:** Comparison of parameters used for calculation of LoG. Upper 4 images: smoothing values of 1, 2.5, 4 or 10. Lower panels: variable thresholds (Fig 2, step 9). Values boxed in green show optima that enable best recognition of anaphase figures. Red pixels in bottom row are above the applied threshold. **D:** Comparison of two local threshold methods to spot neighboring cells after anaphase detection (Fig 2, step 13). This step is challenging because the mCherry::histone 2B signal of neighboring cells varies significantly. Shown is a receiver-operator (ROC) curve comparing default and intermodes threshold calculations methods for 56 micropatterns with detected anaphases amongst ctrl and LGN siRNA conditions (95% confidence interval). An ideal ROC curve should show 100% true positives and 0% false positives (green dashed line). Note that the default threshold method efficiently excludes micropatterns with more than one cell, but also excludes a significant fraction of micropatterns that are properly occupied, thereby decreasing the detection of true positives (Y axis, true positives). Therefore, we used instead the less stringent intermodes threshold method, which increases sensitivity of detection at the cost of including more false positives with more than one cell per micropattern.

In a first "preprocessing" block of TRACMIT, the entire stack of images from the time-lapse recording corresponding to each field of view is aligned using scale invariant feature transform (SIFT) registration [15] to compensate for potential microscope stage drift and positioning hysteresis during acquisition (Fig 2, green boxes, step 1, Fig 3A).

In the second "pattern detection & filtering" block, all micropatterns are detected using their fluorescence signal and characteristic L-shape; micropatterns that are empty or contain more than one cell are excluded (Fig 2, yellow boxes, steps 2 to 7). Note that TRACMIT can detect micropatterns with a different shape following adjustment of parameters using the "Pattern Wizards" (S1 document). The strategy at this stage is to identify each micropattern and to build a “region of interest” bounding box around it. To this end, we use a Minimum Intensity Projection (MinInt) on the SIFT-corrected time-lapse stacks in order to identify L-shaped areas (Fig 2, step 2). MinInt Projections of time-lapse stacks retain shapes that are not moving over time, such as micropatterns in this case (see S2 Movie for illustration). The MinInt image is then smoothened with a median filter and a threshold applied using Huang’s method [16]. To detect the objects on this mask and to filter them based on the expected micropattern area (Fig 2, step 3), it is sufficient to extract the lower left XY position of the L-shape (Fig 2, step 4). From there, a new bounding box is set (Fig 2, step 5) as a region of interest (ROI). The size of this ROI is chosen so as to exclude cells whose nucleus is not positioned sufficiently close to the center between the two arms of the L, since such cells do not stretch properly on the micropattern (data not shown). In parallel to executing steps 2 to 5 shown in Fig 2, a ratio consisting of standard deviation (StDev) of pixel intensities divided by average pixel intensities over 180 frames is calculated (fStDev, Fig 2, step 6 and Fig 3B). Normalizing the absolute standard deviation with the average intensities is needed in order to be independent of variability in mCherry::histone 2B transgene signal. FStDev is a good indicator for cell movement and, therefore, an indirect but robust measure for the number of cells per pattern. This fStDev is measured inside each bounding box and a bandpass cutoff is applied, thereby excluding in a robust manner micropatterns without cells, as well as micropatterns that potentially contained more than one cell (Fig 2, steps 7, 8, Fig 3B). Importantly, restricting further analyses to only those ROIs that contain a single cell helps to reduce the data volume that will be analyzed in the subsequent blocks.

In the third block of TRACMIT, termed “anaphase detection” (Fig 2, dark yellow boxes), a 2D Laplacian of Gaussian (LoG) [17, 18] is calculated for each single frame of the SIFT-aligned stack, resulting in a LoG timelapse (Fig 2, step 9). This enhances small oblong features and increases the contrast between metaphase/anaphase figures and the fluorescent signal of the micropattern itself. We identified optimal settings for this operation empirically as illustrated in Fig 3C. The next goal is to find anaphase figures in this LoG stack. To do so, we apply a hard threshold on the Laplacian stack (Fig 2, step 10), followed by a morphological closing (Fig 2, step 11), which helps correct for interrupted LoG metaphase/anaphase shapes due to fluorescence intensity differences within condensed chromatin. To limit the data volume that needs to be searched for anaphase figures, the boxes from step 7 above are used. Within those boxes, TRACMIT detects pairs of condensed chromosomes that are at a given distance from one another, and share similar angles and areas within a given tolerance (Fig 2, step 12, green boxes). The parameters allowing such detection were optimized manually for HeLa cells during pipeline development until all anaphase figures in randomly selected regions of different wells were detected (see S1 Table for parameters). In order to avoid detecting the same anaphase figure twice at different time points, anaphase figures that are found at the same location in a subsequent time point are ignored. Large differences in mCherry::histone H2B signal intensities were encountered between cells, which resulted in micropatterns containing more than one cell despite the initial bandpass cutoff (Fig 2, step 7). We therefore implemented an additional local threshold at this stage (Fig 2, step 13). We tested two different thresholding methods in order to maximize the number of analyzable micropatterns (see Fig 1B green boxes, exactly one cell per micropattern). The iterative intermeans algorithm (known as the default thresholding method in ImageJ/Fiji) had a higher rate of false negatives (Fig 3D, default), which resulted in the loss of analyzable micropatterns. Although the second method, which assumes a bimodal histogram [19], yielded false positives more readily (see Fig 1B red box “2”, micropatterns with more than one cell; Fig 3D, intermodes), it was preferred to maximize the number of analyzable cells.

In the fourth block termed "prometaphase backtracking" (Fig 2, orange boxes), metaphase plates are detected by tracking back in time from the detected anaphase figures. Metaphase configurations are detected initially as a reunion of the two sets of anaphase chromosomes into one metaphase plate, which implies that the cell underwent successful chromosome segregation (Fig 2, step 15). Such detection is achieved by checking frame by frame if the intensities of a line profile between the two sets of anaphase chromosomes fall below a certain threshold on the LoG image. This is interpreted as the reunion of the anaphase figures into a metaphase plate. This metaphase plate now serves as a seed for backtracking until prometaphase (Fig 2, step 16). A gap of three frames is allowed whilst doing so in order to account for potential errors in segmentation or filtering; objects corresponding to chromosomes are then linked together over time based on an extended Brownian motion tracker. We consider that the metaphase plate preceding the anaphase figure is detected if it is the closest object from the position of the last tracked anaphase figures, and if it has an empirically defined area and shape (fitted ellipse major/minor axis sizes and ratios, see S1 Table).

The fifth and last block of TRACMIT is "export results" in which the most relevant data is computed and exported (Fig 2 pale blue box). In the case of the spindle positioning screen, this corresponds to the angle of the metaphase spindle with respect to the horizontal arm of the L-shape, as well as the duration of metaphase. Importantly, in addition, a simple interface has been designed to allow the user to manually visit any detection and thus verify the successful spotting of relevant metaphase figures (S1 document).

### Pipeline validation and performance

**Fig 4:**
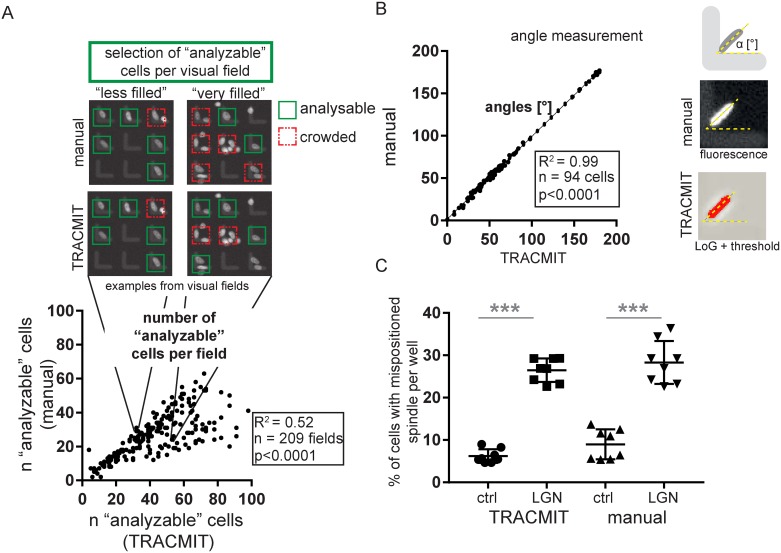
TRACMIT validation and performance. **A:** Correlation of “analyzable” cells detected per well (n), comparing manual analysis with TRACMIT; 209 siRNA conditions (wells), >9000 cells in total. Shown are high magnification views from plates with "less filled" and "very filled" conditions, extracted from the graph below, as indicated. Pearson correlation coefficient, R^2^ = 0.52, p<0.0001. **B:** Correlation of angle measurements between manual analysis and TRACMIT. Pearson correlation coefficient, R^2^ = 0.99, p<0.0001. **C:** Proof of principle: spindle positioning phenotype (= % of cells with mispositioned spindle per well, i.e. a metaphase plate with an angle >40° off from the normal 45° position) in wells treated with control (ctrl) or LGN siRNA, comparing manual analysis and automated analysis with TRACMIT. N = 8 wells per condition (15–40 cells per well). Two tailed Student’s t-test *** p<0.001.

We validated the different stages of TRACMIT as follows. First, to estimate the rate of false positive detection, we manually inspected 9045 cells from 209 different siRNA conditions imaged during the screen (Wolf et al., manuscript in preparation), which TRACMIT deemed to be analyzable. As shown in Fig 4A, we found a Pearson correlation coefficient R^2^ of 0.52 between TRACMIT and manual inspection, primarily due to the pipeline not recognizing some micropatterns that had more than one cell (Fig 4A, skew towards the bottom right), which was anticipated given the choice of the intermodes threshold step (Fig 2, step 13 and Fig 3D).

We next analyzed the accuracy of metaphase spindle angle determination by TRACMIT compared to manual measurements. To this end, we randomly selected 94 cells that had been analyzed by TRACMIT and performed manual angle measurements on the corresponding raw images (Fig 4B). Importantly, we found an almost perfect correlation between the two approaches (R^2^ = 0.99, Fig 4B).

Thereafter, we asked whether despite the relatively high rate of false positive detection (see Fig 4A), TRACMIT could perform as accurately as an individual in discriminating between a control condition and a condition exhibiting a spindle positioning phenotype. To this end, we compared a control condition (ctrl siRNAs) with a condition known to exhibit a spindle positioning phenotype (siRNAs against LGN); a cell was deemed to exhibit a spindle positioning phenotype whenever the metaphase plate had an angle > 40° off from the normal 45° position. Importantly, as shown in Fig 4C, we found that TRACMIT performed as well as a human being in recognizing wells with a high percentage of cells exhibiting a spindle positioning phenotype.

## Conclusion

We provide a tool for automated chromatin feature extraction of mitotic cells grown on micropatterns, thus allowing screening of live imaging data sets for novel candidates regulating mitotic processes, such as spindle positioning. This fully automated pipeline is uniquely tailored to solve challenges faced in such assays, including selecting micropatterns that contain exactly one cell, while incorporating pre-processing, tracking, data filtering and visual validation. In summary, TRACMIT provides a new means to monitor and quantify cell division features in an efficient and open source manner.

## Materials and methods

Further description of the actual spindle-positioning screen will appear elsewhere (Wolf et al., manuscript in preparation).

### Cell culture, reverse transfection with siRNA and cell plating on micropatterns

HeLa mCherry::histone 2B cells (gift from Arnaud Echard, Institut Pasteur, Paris) were cultured in high-glucose DMEM with GlutaMAX (Invitrogen) media supplemented with 10% FCS in a humidified 5% CO2 incubator at 37°C. For siRNA experiments, ∼60,000 cells per well were seeded on 96-well cell culture plates (Greiner, plastic, flat bottom) and transfected with 20nM validated stealth siRNAs against LGN (sequence: UAGGAAAUCAUGAUCAAGCAA, Qiagen) or scrambled siRNA (SI03650318, Quiagen) for 48h. Cells were then detached using 40ul Accutase per well (Sigma, A1110501 XY) and transferred to custom-designed 96-well plates containing L-shaped micropatterns (area: 700 μm^2^, 6000 patterns per well, 20μm distance between patterns) coated with fibronectin coupled to Alexa Fluor 555 (Cytoo). 6000 cells were transferred into each well. After letting cells attach to the micopatterns for up to 6 hours, the plates were transferred to the microscope for imaging.

### Live imaging and data storage

Micropatterned 96-well plates were imaged for 24 hours using a GE InCellAnalyzer 2200 microscope equipped with a sCMOS CCD camera, at 10x magnification (NA 0.45 Plan Apo), 37°C, 5% CO2. Each field of view was imaged in a single focal plane every 8 minutes with an exposure time of 100ms, capturing 2 fields of view per well, using hardware autofocus and a LED transmitted light source. Voxel size was 0.65 μm and fluorescence intensities ranged from 250 to 4000 AU (see Fig 1C). The imaging data (400 Gb per 96-well plate, 16-bit image stacks) was stored on a local server as well as on SATA drives (WD, 2.0 TB, SATA, 64MB Cache), which were used for analysis.

### Programming

We programmed TRACMIT in ImageJ macro language. The code is deposited on GitHub and includes installation instructions as well as a user’s guide.

## Supporting information

S1 Movie Movie of magnified inset from Fig 1B, filmed over 24h (180 frames, 8 minutes framerate, 24 hours in total); micropatterns are labelled with Alexa Fluor 555 and HeLa cells express mCherry::histone 2B. (AVI)

S2 Movie Demonstration of pattern detection using Minimum Intensity projection using SIFT-corrected S1 Movie as raw data. (AVI)

S1 Table TRACMIT settings for HeLa cells. (PDF)

S1 document TRACMIT user guide. (DOCX)

S2 document TRACMIT workflow availability and requirements. (DOCX)

## Abbreviations

Alexa 555: Alexa Fluor 555 fluorescent dye
Ctrl: negative ctrl, scrambled siRNA
HeLa: cervical cancer cell line, derived from Henrietta Lacks
LAP: linear assignment problem
LGN: protein containing leu-gly-asn (LGN) repeats
LoG: Laplacian of Gaussian
L-shape: micropattern that is shaped like the letter L, with both arms of the L having the same length
MinInt: minimum intensity
ROI: region of interest
SIFT: scale invariant feature transform
TRACMIT: pipeline for TRACking and analyzing cells on micropatterns through MITosis
