# Supplementary material for: TRACMIT: An effective pipeline for tracking and analyzing cells on micropatterns through mitosis
Source: PLoS One. 2017 Jul 26;12(7):e0179752. doi: 10.1371/journal.pone.0179752 (PMC5528263; doi:10.1371/journal.pone.0179752)
Supplement: S1 Table — (PDF) [file pone.0179752.s003.pdf]

Supplemental Table 1

Pseudo Flatfield Blur	30
Pattern Mask Median Filter	6
Pattern Min Area	350
Pattern Max Area	Infinity
Pattern Min SD (ratio Av/SD)	0.1
Pattern Max SD	0.45
Laplacian Smoothing	2.5
Laplacian Threshold	-40
DNA Min Area	10
DNA Max Area	80
Division Area Tolerance	10
Division Angle Tolerance	15
Division Max Distance	25
Max Frame until first mitotic plate	2
ROI threshold	Intermodes
Mitotic Plate Minimal Laplacian	-60
Max Total Area of Single Cell	190
Min Single Cell Area	20
Mitotic Plate Min Area	30
Mitotic Plate Max Area	130
Mitotic Plate Min Major Axis	6
Mitotic Plate Max Major Axis	25
Mitotic Plate Min Minor Axis	2
Mitotic Place Max Minor Axis	6.8
Mitotic Plate Min Axis Ratio	2.9
Mitotic Plate Max Axis Ratio	6
Mitotic Plate Max Movement	5
Mitotic Plate Max Frames to seek	3
