# Supplementary material for: TRACMIT: An effective pipeline for tracking and analyzing cells on micropatterns through mitosis
Source: PLoS One. 2017 Jul 26;12(7):e0179752. doi: 10.1371/journal.pone.0179752 (PMC5528263; doi:10.1371/journal.pone.0179752)
Supplement: S1 document — (DOCX) [file pone.0179752.s004.docx]

TRACMIT

User Guide

TRACMIT 1.1

<https://github.com/lacan/TRACMIT>

Contents

Installation 3

Dependencies 3

Using Fiji Update Sites 3

Manual Installation 3

Sample Dataset 3

Interface 4

TRACMIT User Interface 4

TRACMIT Settings Bar 5

Full Interface when running 6

Regions of Interest Manager 6

Image Calibration 7

Parameter Optimization 8

Using the Parameter Wizards 8

Fine-Tuning the Parameters 9

Example Use 10

Troubleshooting 11

# Installation

## Dependencies

As you complete the installation steps below, make sure that the following update sites are enabled:

- **IBMP-CNRS** Contains the ActionBar Plugin by Jerôme Mutterer.
- **PTBIOP** Contains the BIOPLib and attached plugins used for managing. TRACMIT's settings and other internals.
- **Imagescience** Contains the FeatureJ Laplacian Plugin used by TRACMIT.

## Using Fiji Update Sites

The simplest way to install TRACMIT is to use the TRACMIT Update site through Fiji:

1. From Fiji, go to **Help > Update...**
2. Select **Manage Update Sites.**
3. Click on **Add Update Site**, this will create a new line on the table.
4. Change the Name to "**TRACMIT**", for clarity's sake.
5. In the URL column, enter or paste [**http://biop.epfl.ch/TRACMIT/**](http://biop.epfl.ch/TRACMIT/)
6. Click on **Close.**
7. Finally click on **Apply Changes** and **restart Fiji.**
8. After these steps, you should find **TRACMIT under Plugins > ActionBar.**

## Manual Installation

We do not recommend manual installation as TRACMIT depends on multiple packages that would become difficult to manage outside of the Fiji Update Site solution.

## Sample Dataset

To test TRACMIT, you can download a sample dataset from ZENODO with the following DOI:

<https://doi.org/10.5281/zenodo.232218>

# Interface

##
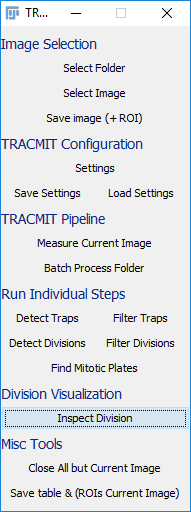
TRACMIT User Interface


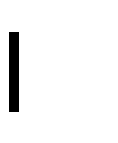

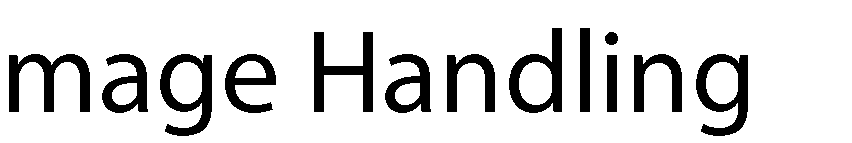

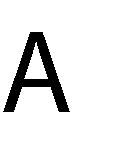

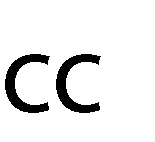

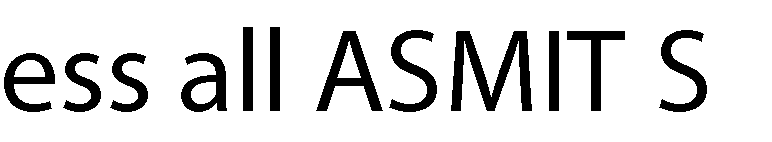

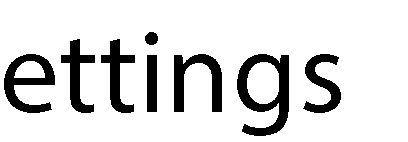

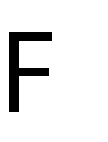

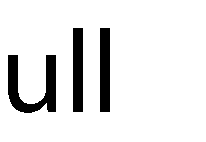

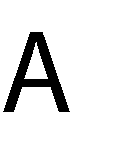

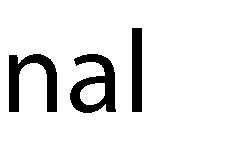

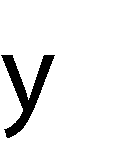

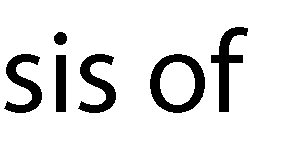

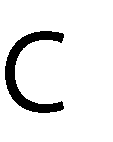

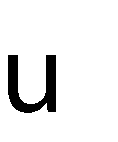

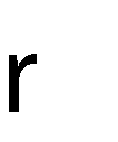

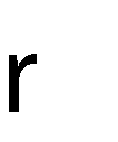

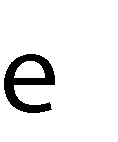

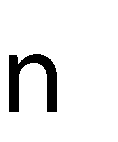

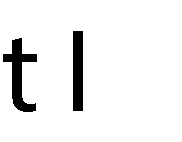

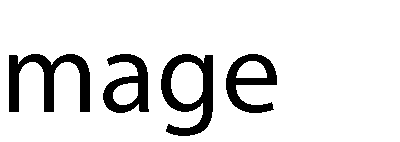

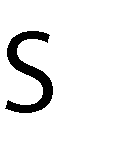

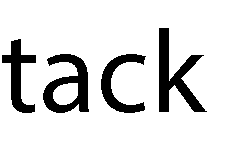

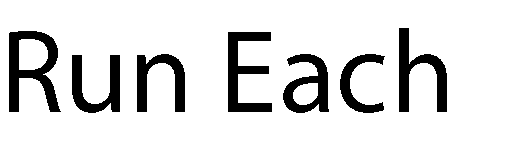

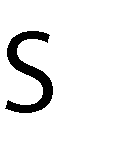

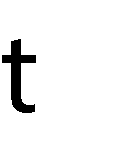

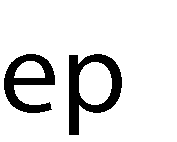

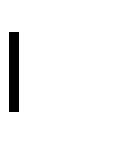

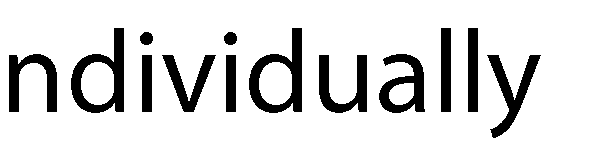

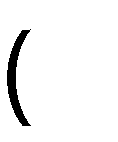

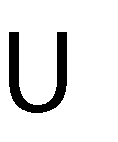

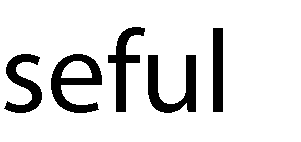

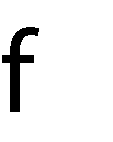

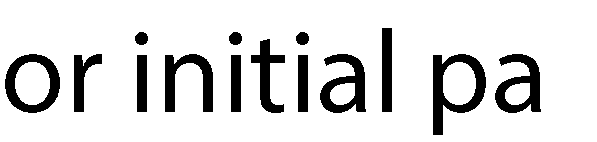

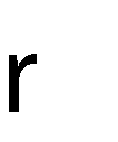

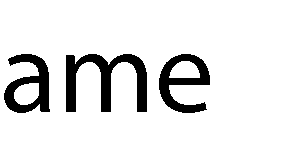

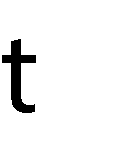

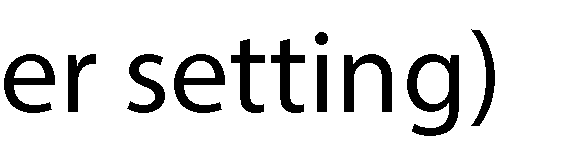

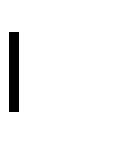

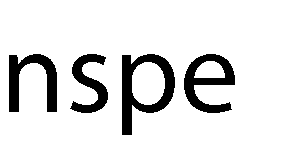

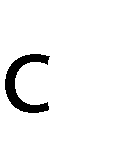

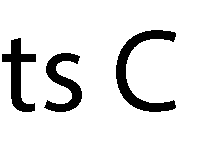

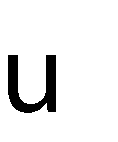

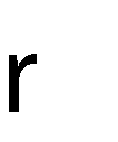

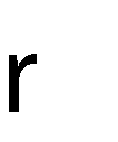

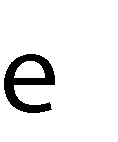

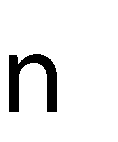

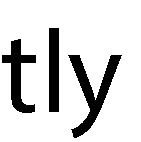

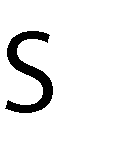

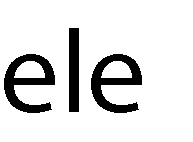

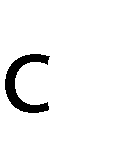

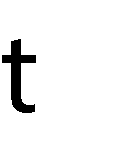

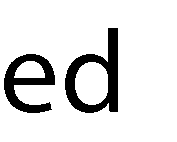

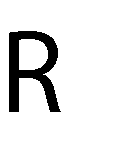

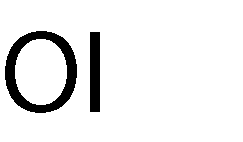

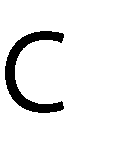

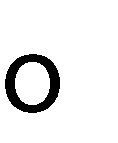

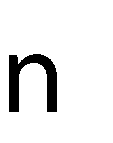

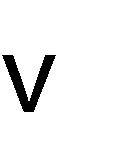

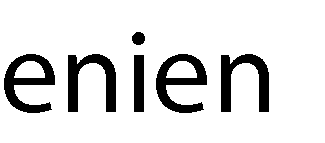

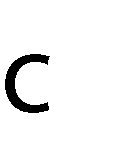

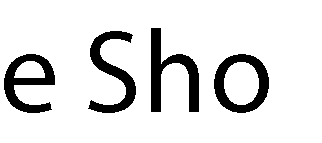

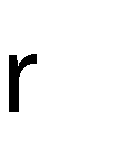

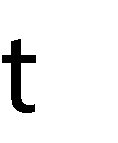

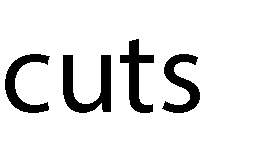


## TRACMIT Settings Bar
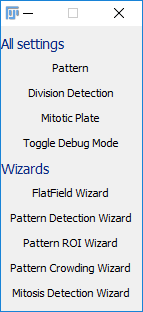


Wizards for setting optimization

Use these to get an initial estimate of TRACMIT’s Parameters.

All settings relevant to TRACMIT

Use these to fine-tune your parameters.

## Full Interface when running


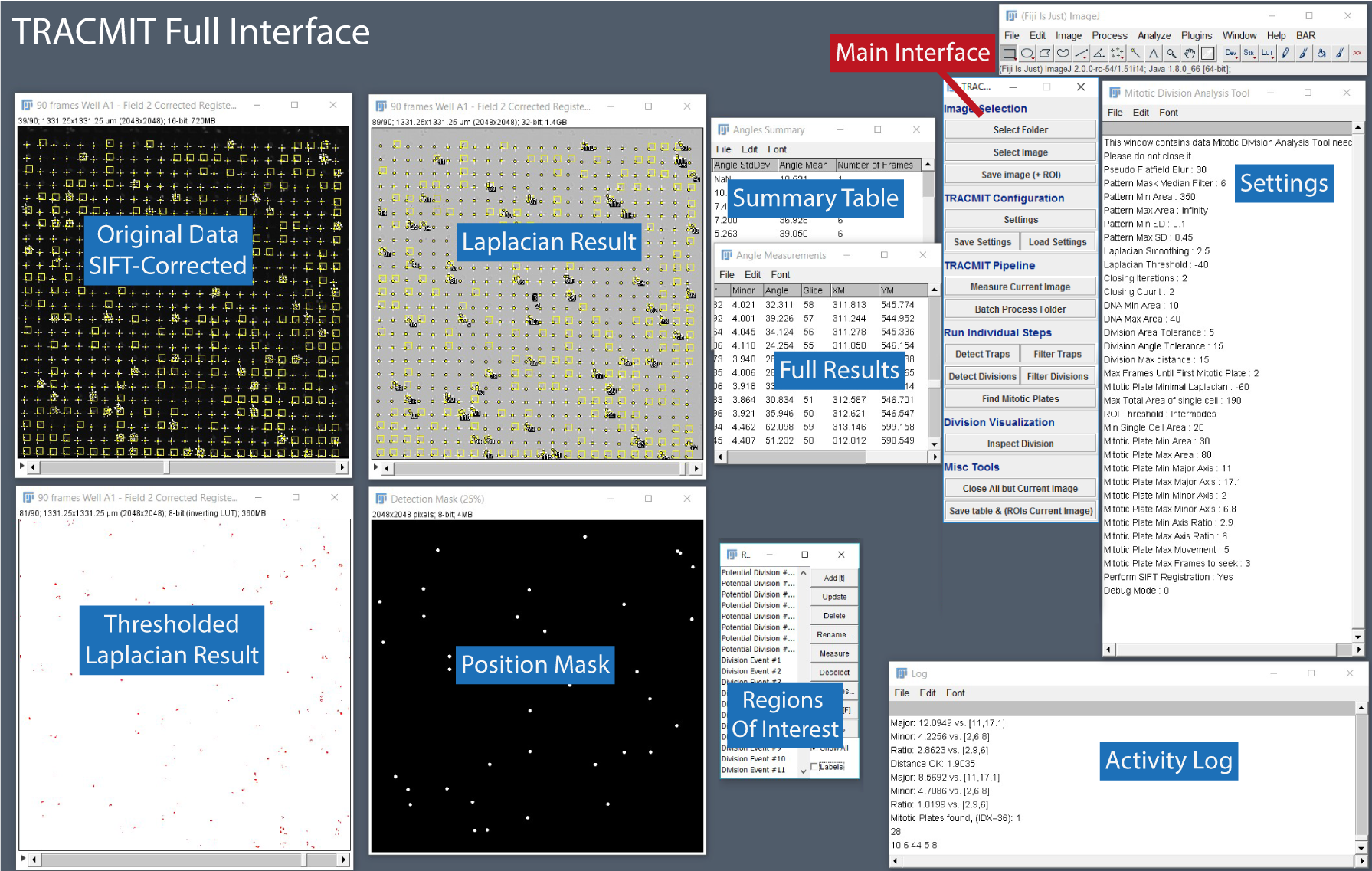


When TRACMIT is running, several Windows work together as shown above.

- **Original Data** contains the SIFT-Corrected original dataset loaded with Select Image or Select Raw Image.
- **Laplacian Result** is the 2D Laplacian of Gaussian timelapse used throughout TRACMIT.
- **Thresholded Laplacian Result** contains the binarized Laplacian Image used for extracting shape features.
- **Position Mask** helps TRACMIT check if a division event was already detected at a certain location, to avoid duplicate detections.
- **Full Results** contains all the data on the detections, for each detection event in each frame.
- **Summary Table** is also available, with one line per division event.
- **Regions Of Interest** is the ROI Manager of ImageJ with all detections named (See [Regions of Interest Manager](#_bookmark8) section).
- **Settings** contains a list of all the variables used by TRACMIT[.](#_bookmark8)
- **Activity Log** helps the user follow the progress of TRACMIT.

##
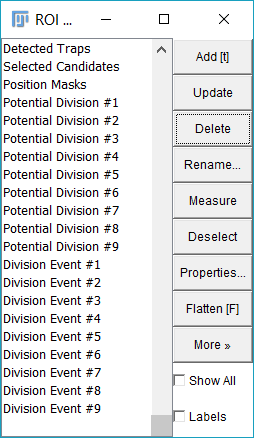
Regions of Interest Manager

At the end of processing, the ROI Manager contains 5 Types of ROIs:

- **Detected Traps**: a series of points that should match the bottom left of the patterns (see main publication, Figure 2 Step 5).
  - **Parameters used**: Pattern Mask Median Filter, Pattern Min Area, Pattern Max Area.
- **Selected Candidates**: a series of points that represents the patterns kept after Standard Deviation Filtering (see main publication, Figure 2 Step 8).
  - **Parameters used:** Pattern Min SD, Pattern Max SD.
- **Position Masks**: an ROI showing where the divisions will be searched for within the stack (see main publication, Figure 2 Step 8).
- **Potential Division #X**: Each ROI consists of two points that indicate the objects that were interpreted by TRACMIT as being anaphase figures (see main publication, Figure 2 Step 12). You can visit them by clicking on the **Inspect Division** Button.
  - **Parameters used:** DNA Min Area, DNA Max Area, Division Area Tolerance, Division Angle Tolerance, Division Max Distance, ROI threshold, Max Total Area of Single Cell, Min Single Cell Area.
- **Division Event #X**: Point ROI showing the stack position of a division event in which a metaphase plate was found and backtracked. You can visit each division event by selecting the desired ROI and clicking on the **Inspect Division** Button (see main publication, Figure 2 end of step 16)
  - **Parameters used**: Max Frame until first mitotic plate, Mitotic Plate Minimal Laplacian, Mitotic Plate Min Area, Mitotic Plate Max Area, Mitotic Plate Min Major Axis, Mitotic Plate Max Major Axis, Mitotic Plate Min Minor Axis, Mitotic Place Max Minor Axis, Mitotic Plate Min Axis Ratio, Mitotic Plate Max Axis Ratio, Mitotic Plate Max Movement, Mitotic Plate Max Frames to seek.

# Image Calibration

We highly recommend users to have calibrated images (i.e. images with a known pixel size, usually in microns). In this manner, all parameters in TRACMIT will be in calibrated units, and thus be more readily understandable for the users, independently of the objective that was used.

**Warning**: The only value that is not calibrated is the Laplacian sigma, which remains in pixels.

To check whether your image is calibrated, you can go to Image > Properties from the Fiji main window and have a result similar to the figure below on the left.


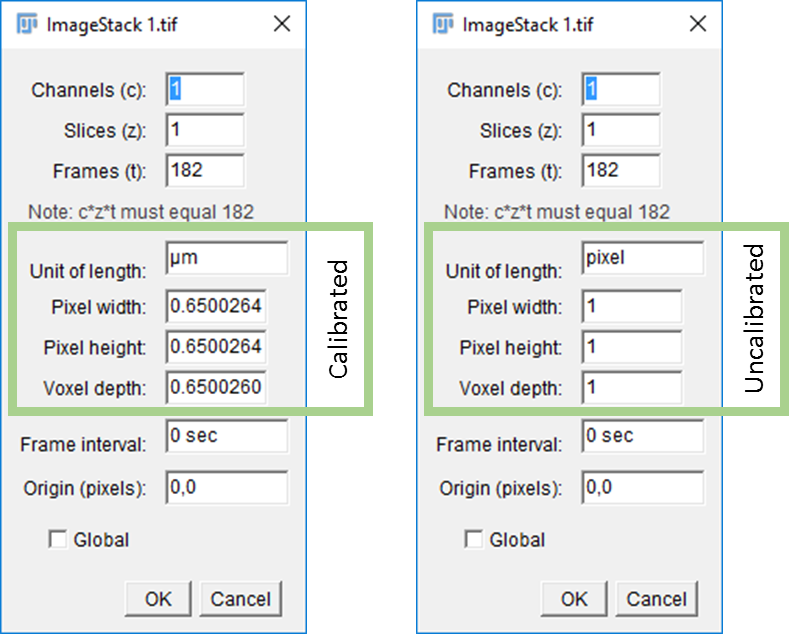


In case the images are not calibrated, the user can obtain the calibration information in several ways.

1. Loading images from native microscope formats using BioFormats usually results in calibrated images.
2. Using a micro-ruler to calibrate an image taken with the same optical configuration, see https://imagej.net/SpatialCalibration.
3. If all optical elements are known, the following formula can be used to estimate the pixel size:


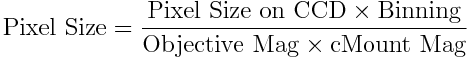


# Parameter Optimization

## Using the Parameter Wizards

The Parameter Wizards provide a simple interface to tune TRACMIT’s parameters to a new dataset.

It is highly recommended to initially work on a small subset of your data. Choose regions in your data where division events are taking place and crop them out in XY and in time. This will make the initial parameter estimation and subsequent fine-tuning faster and more convenient.

In our publication, the initial datasets were 2048x2048 in size and there were 182 timepoints. Cropped versions were about 512x512 across 182 timepoints.

### FlatField Wizard

You are prompted to draw a rectangular region that corresponds to the largest structure that you would like to keep on your image. Usually, this is the size of a full micropattern if present, or a couple of cells long at least. This sets the **Pseudo Flatfield Blur** as the longest dimension of the drawn rectangle.

### Pattern Detection Wizard

This tool segments out the patterns and asks the user to select in the ROI manager (Holding the CTRL key) some representative patterns in order to estimate the pattern area ranges that TRACMIT will use to filter patterns from noise.

This sets the **Pattern Min Area** and **Pattern Max Area** as the mean area of the selected patterns minus/plus 3 times the standard deviation, respectively.

### Pattern ROI Wizard

To accommodate different patterns and geometries, this wizard averages all patterns that were detected using the criteria from the Pattern Detection Wizard.

It then prompts the user to draw a rectangle on the Average Pattern image. This pattern should represent the area inside which one expects mitosis to happen. The user is then shown what that region looks like on the detected pattern Mask.

This sets the **Division Detection Box Width, Division Detection Box Height, Division Detection Box X offset, Division Detection Box Y offset, Pattern Detection Box Width, Pattern Detection Box Height**

### Pattern Crowding Wizard

Patterns with too many or too few cells are identified using a Standard Deviation Projection of the time stack. With this wizard, the user is prompted to select (Holding the CTRL key) the ROIs (calculated during the Pattern Detection Wizard) that have a single cell on them. Based on this selection, the wizard will suggest initial settings for the **Pattern Min SD** and **Pattern Max SD** as 0.7x/1.3x the computed minimum and maximum values of the selected regions, respectively.

Finally, the wizard will run the mitotic plate detection and prompt the user to instruct the wizard if the currently highlighted trap represents a crowded pattern or not. This will set the **Max Total Area of single Cell** as 1.2x the largest area the user determined as belonging to a pattern with a single cell.

### Mitosis Detection Wizard

The wizard prompts the user to draw a line along the **major** axis of a metaphase plate that is going to divide in the next two frames. Using this line, the wizard then fits the data and calculates the metaphase plate size along the major and minor axes. It uses the fact that two frames later there is an anaphase figure to estimate the acceptable Area range, the initial Laplacian of Gaussian, the Laplacian Threshold, the DNA Min and Max Areas, the Major and Minor Axis extents of the Mitotic plates and the max movement allowed.

This wizard helps to find initial values for all the parameters below.

**Laplacian Smoothing** as half of the full width at half maximum of the minor axis.

**Laplacian Threshold** as 0.2x of the minimum value of the line profile along two consecutive anaphase plates.

**DNA Min Area** as 0.7x of the average area of each individual anaphase figure detected with the given Laplacian smoothing and threshold determined above.

**DNA Max Area** as 1.3x of the average area of each individual anaphase figure detected with the given Laplacian smoothing and threshold determined above.

**Division Max distance** as 1.2x the inter anaphase figure distance computed from the detections above.

**Mitotic Plate Minimal Laplacian** as 1.3x the minimum value of the mitotic plate detected using the given smoothing and thresholds.

**Mitotic Plate Min Area** as 0.7x the value of the mitotic plate area detected using the given smoothing and thresholds.

**Mitotic Plate Max** Area as 1.3x the value of the mitotic plate area detected using the given smoothing and thresholds.

**Mitotic Plate Min Major Axis** as 0.7x the value of the full width at half maximum of the major axis computed from the line profile.

**Mitotic Plate Max Major Axis** as 1.3x the value of the full width at half maximum of the major axis computed from the line profile.

**Mitotic Plate Min Minor Axis** as 0.7x the value of the full width at half maximum of the minor axis computed from the line profile.

**Mitotic Plate Max Minor Axis** as 1.3x the value of the full width at half maximum of the minor axis computed from the line profile.

**Mitotic Plate Min Axis Ratio** as 0.7x the ratio of the major and minor axes full width at half maximums computed from the line profile.

**Mitotic Plate Max Axis Ratio** as 1.3x the ratio of the major and minor axes full width at half maximums computed from the line profile.

**Mitotic Plate Max Movement** as 3x the xy voxel size

## Fine-Tuning the Parameters

As the interface provides a way to perform each step individually, you can optimize parameter sets independently. Refer to the section above to see which parameters are used in which step.

In order to find the values that would best match the data, such as Min/Max Areas of mitotic plates, the simplest approach is as follows:

1. Locate mitotic plates manually within the **Thresholded Laplacian Result** image.
2. Use the ImageJ Magic Wand tool to select a metaphase plate.
3. Hit “M” which will measure the current selection and give you the values associated with that metaphase plate.
4. Repeat for multiple observations and at different timepoints, and you will obtain a table from which you can infer the min and max values to use.

The above procedure works for most parameters.

# Example Use

To run our example dataset, proceed as follows:

- 1. Download the default parameters (TRACMIT Default Settings.txt) for this dataset from <https://github.com/lacan/TRACMIT>
  2. Download the sample dataset from <https://doi.org/10.5281/zenodo.232218>.
  3. Extract the dataset ZIP file.
  4. Launch TRACMIT from Plugins > ActionBar > TRACMIT 1.1.
  5. Click on **“Load Parameters”** and use the downloaded txt file.
  6. Click on **“Select Image”.**

You will be prompted to provide the location of the extracted folder.

- 1. It will offer you two fields, open whichever one you would like to analyze.
  2. Click on **“Measure Current Image”.**
  3. The results tables might be located behind an empty results table. Make sure that you move it to see it…
  4. You can inspect the detected divisions by highlighting the one you would like to see from the ROI Manager and clicking on **“Inspect Division”.**

# Troubleshooting

Feel free to report potential issues and to ask questions on TRACMIT’s GitHub Page:

<https://github.com/lacan/TRACMIT/issues>
