# Supplementary material for: TRACMIT: An effective pipeline for tracking and analyzing cells on micropatterns through mitosis
Source: PLoS One. 2017 Jul 26;12(7):e0179752. doi: 10.1371/journal.pone.0179752 (PMC5528263; doi:10.1371/journal.pone.0179752)
Supplement: S2 document — (DOCX) [file pone.0179752.s005.docx]

# Workflow availability and requirements

Project name: TRACMIT

Project home page: https://github.com/lacan/TRACMIT

Operating systems(s): Agnostic

Programming language: ImageJ, JAVA based

Other requirements: ImageJ/ Fiji

Any restrictions to use by non-academics: none
